# Possible therapeutic effect of royal jelly on endometriotic lesion size, pain sensitivity, and neurotrophic factors in a rat model of endometriosis

**DOI:** 10.14814/phy2.15117

**Published:** 2021-11-21

**Authors:** Zahra K. Farahani, Mahnaz Taherianfard, Mohamad Mehdi Naderi, Hortensia Ferrero

**Affiliations:** ^1^ Physiology Division of Basic Sciences Department School of Veterinary Medicine Shiraz University Shiraz Iran; ^2^ Maternal, Fetal, and Neonatal Research Center Tehran University of Medical Sciences Tehran Iran; ^3^ Reproductive Biotechnology Research Center Avicenna Research Institute ACECR Tehran Iran; ^4^ Institute of Treatment and Diagnosis of Uterine Diseases IVI Foundation Valencia Spain

**Keywords:** endometriosis, lesion size, neurotrophic factor, pain, royal jelly

## Abstract

Endometriosis is the abnormal growth of endometrial tissue. The goals of the study are: (1) Is any correlation between endometriosis pain and neurotrophins in the serum, dorsal root ganglion (DRG), and peritoneal fluid (PF) in rat models of experimental endometriosis?, (2) Possible therapeutic effects of royal jelly (RJ) on pain scores, size of endometriotic lesion, and neurotrophic factors. Forty‐eight Sprague Dawley female rats weighing 205.023 ± 21.54 g were maintained in a standard condition. The rats were randomly divided into one of the six groups: Control (no intervention), Sham‐1 (remove of uterine horn), RJ (administration of 200 mg/kg/day RJ for 21 days), Endometriosis (induction of endometriosis), Treatment (induction of endometriosis+administration of 200 mg/kg/day RJ for 21 days), and Sham‐2 (induction of endometriosis+administration of water). Formalin test performed for pain evaluation. The levels of Brain‐derived neurotrophic factor (BDNF), nerve growth factor (NGF), substance P, and calcitonin gene‐related peptide (CGRP) were measured by enzyme‐linked immunosorbent assay. The mean pain scores in all three phases of the formalin test were significantly increased by endometriosis induction (*p *< 0.05). The concentrations of BDNF, NGF, and CGRP in DRG of the endometriosis group were significantly higher than these factors in the Control, Sham‐1, and RJ groups (*p* < 0.05). RJ could significantly (*p *< 0.001) decrease the mean lesion size and the mean pain score in the late phase (*p *< 0.05). The present results determine that endometriosis pain may be related to nervous system neurotrophic factors. Treatment with RJ could decrease the size of endometriosis lesions as well as pain scores. The findings may shed light on other complementary and alternative remedies for endometriosis.

## Graphical abstract

Treatment with Royal Jelly could decrease the size of endometriosis lesions as well as pain scores.

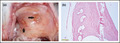

## 1 INTRODUCTION

Endometriosis is defined as the presence of the endometrial gland and stroma in extra‐uterine sites. It has a prevalence of 15%–25%; however, it is seen in about 50% of the infertile women (Cheng et al., 2017; Foti et al., 2018; Zhang et al., 2017). Although pain is the most common complaint, the exact pain mechanisms are still unclear. Numerous studies explained the possible mechanisms of pain in endometriosis (Coxon et al., 2018; Lian et al., 2017). It has been shown that endometrial mucosal tissue does not have high sensory innervations; however, a high density of these fibers is present in the endometriotic lesions (Greaves et al., 2017; Miller & Fraser, 2015). Several studies have found that an increase in inflammatory responses, including prostaglandin E2 (PGE2)‐signaling pathway, transient receptor potential cation channel subfamily V member 1 (TRPV1) in nociceptive neurons, and pain‐related neurotransmitters like calcitonin gene‐related peptide or substance P in neuronal circuits resulting in pain (Schou et al., 2017; Tokushige et al., 2006).

Recently, a limited number of investigations has pointed to the role of neurotrophins (NTs) in endometriosis pain. It seems that changes in NTs and NT receptors can induce hyperalgesia. NTs belong to the growth factor family and cause neurogenesis. So far, several types of NT proteins have been discovered. Brain‐derived neurotrophic factor (BDNF), nerve growth factor (NGF), NT 3, and NT 4 are the most important ones (Arellano et al., 2013; Zhang et al., 2008). The NTs over‐expression causing the increase of chronic pelvic pain was confirmed in endometriosis patients (Kobayashi et al., 2014). Stefani et al. (2019) found a higher serum concentration of BDNF in patients with chronic pain problems. An increase of BDNF levels was shown in the serum and peritoneal fluid of endometriosis women by Ding et al. (2018); however, this increase was higher in painful endometriosis compared to endometriosis without pain. It also reported that assessment of plasma BDNF could be implemented as a clinical biomarker for endometriosis pain (Rocha et al., 2017; Wessels et al., 2015). On the other hand, the reliable predictive power, sensitivity, or specificity of this marker was not confirmed by Perricos et al. (2018). Morotti et al. (2014) did not show a clear link between these factors and endometriosis pain. Regarding NGF expression in the PF, Arellano et al. (2011) found no significant differences in the endometriosis patients with different pain scores. So, they concluded that the neurotrophic factors of the endometriotic lesion are not pain associated.

Many studies have revealed the antioxidant, anti‐tumoral, anti‐inflammatory, analgesic, and neuronal protective effects of royal jelly (RJ) (Arzi, Houshmand, et al., 2015; Arzi, Olapour, et al., 2015; Aslan & Aksoy, 2015; Kaynar et al., 2012; Teixeira et al., 2017). For instance, an investigation has shown RJ by its antiinflammatory effects (decreasing free‐radical and oxidative stress) could prevent urolithiasis and its inflammation (Aslan & Aksoy, 2015). The other animal study indicated that RJ by increasing antioxidant enzyme activity and decreasing lipid peroxidation could improve methotrexate‐ induced intestinal mucositis in rats (Kaynar et al., 2012). It was reported that these therapeutic effects of RJ may relate to its phenolic compounds such as flavonoids, phenolic acids, 10‐hydroxydecanoic acid, and adenosine monophosphate (Arzi, Houshmand, et al., 2015; Arzi, Olapour, et al., 2015; Aslan & Aksoy, 2015; Kaynar et al., 2012). According to these characteristics, it is supposed that royal jelly may also have positive effects on endometriosis. On the other hand, several studies have shown the estrogenic effects of royal jelly, which may improve the pain of this estrogen‐dependent disease (Bachmann & Nevadunsky, 2000; Mishima et al., 2005; Seyyedi et al., 2016).

These paradoxical findings indicate the need for further research. In addition, to the best of our knowledge, there is no evidence for the possible therapeutic effect of royal jelly on endometriosis and NT alterations. Therefore, the present study was carried out to assess: (1) the possible correlations between endometriosis pain and neurotrophic factors in the dorsal root ganglion (DRG), serum, and peritoneal fluid (PF) in a rat model of endometriosis and (2) the possible therapeutic effects of royal jelly (RJ) on the pain scores, endometriotic lesion size, and neurotrophic factors.

## 2 MATERIAL AND METHODS

The present study was conducted according to the Institutional Research Ethics Committee guidelines of Shiraz University regarding the care and use of laboratory animals in experimental studies.

### 2.1 Study design and animals

Forty‐eight Sprague Dawley female rats weighing 205.78±25.45 g were used. The animals were kept in a standard condition (20–24°C and 12‐h dark‐light cycles). The rats were monitored for a week regarding acclimatization to the new environment and their health conditions. Then, they were randomly divided into one of these six groups in two phases:

#### 2.1.1 Phase one (21 days)

‐ Control group (intact rats).
‐ Endometriosis group (endometriosis induction was performed).
‐ Sham‐1 group (laparotomy was done without endometriosis induction).
‐ Royal Jelly group (200 mg/kg/day of RJ was administered by oral gavage).

#### 2.1.2 Phase two (42 days)

‐ Treatment group (RJ was administered 21 days after endometriosis induction and continued for 3 weeks).
‐ Sham‐2 group (Water was administered 21 days after endometriosis induction and continued for 3 weeks).

**FIGURE 1:**
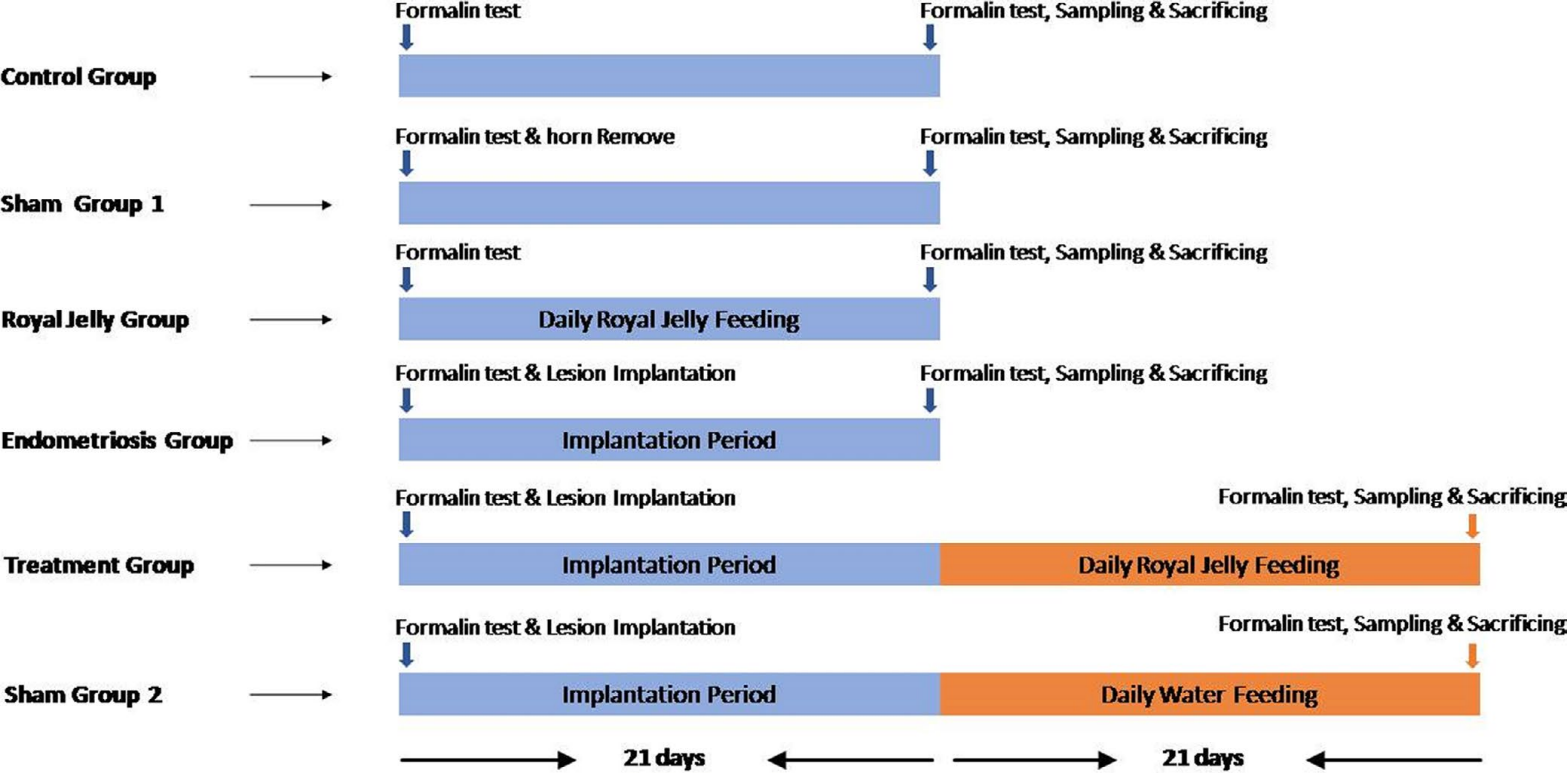
Experimental design paradigm

The formalin test was carried out for each group (8 rats per group) on the first and last days of the study. The test was performed by a colleague who was blind regarding the groups. Figure 1 presents the experimental design.

### 2.2 Formalin test

Pain sensitivity for 60 min was evaluated after formalin injection (50 µl, 2.5%) in the hind paw. The early, intermediate, and late pain responses recorded during 0–5, 6–15, and 15–60 minutes, respectively (Hadi Chegeni et al., 2015; Pirzadeh et al., 2019; Taherianfard & Mosavi, 2011).

### 2.3 Endometriosis induction

Endometriosis induction was carried out as follows. Estradiol 0.2 mg/kg 17β (E8875; Sigma‐Aldrich) was subcutaneously injected on the first day in the estrous phase. The rat was anesthetized using IP injection of xylazine 2% and ketamine 10% (Alfasan Woerden). Skin preparation (prep and drape of the abdominal area) was done carefully. The left uterine horn was removed through an abdominal incision and put in a Petri dish containing PBS. Then, it was opened longitudinally and incised into two 5 × 5 mm slices. Each segment was sutured near the main arteries inside the abdominal wall (using nonabsorbable suture 6‐0). Using nonabsorbable 4‐0, the abdominal wall and the skin were sutured. The estradiol and gentamicin were injected for three consecutive days (Farahani et al., 2020; Zhixing et al., 2015).

The second abdominal surgery was performed for all rats with endometriosis induction (after 21 days for the phase one groups and after 42 days for the phase two groups) (Jouhari et al., 2018). Under deep anesthesia, the abdominal cavity was opened, using a Digital Quails, the dimensions of the endometriotic lesion were measured, and according to the formula, the lesions sizes were calculated (Rudzitis‐Auth et al., 2016):

$$S=\;\text{D1}\;\left(\text{the largest diameter}\right)\times \text{D2}\;\left(\text{perpendicularly aligned diameter}\right)\times \pi /4$$

The lesion was totally removed and for histological confirmation was sent to the Department of Pathobiology.

### 2.4 Sample preparation

On the last day of the study through a deep anesthesia, 2 ml of the cardiac blood was collected. Also, collecting peritoneal fluids, 2 ml warm PBS was injected and aspirated from the abdominal cavity. Finally, the vertebral column was removed and the spinal cord was exposed after euthanizing the animal. DRGs from T13‐L6 (receiving input from pelvic and visceral organs) were removed (do Amaral et al., 2009; Li et al., 2008; Wen et al., 2015). The samples were centrifuged and stored at −70°C.

### 2.5 BDNF, NGF, substancE P, and CGRP measurements

Neurotrophic factors were assessed using the ELISA method according to the ELISA kit brochures. Serum, DRG, and peritoneal fluid were placed in the refrigerator to defrost. After ultrasonic homogenization of DRG, the supernatant was collected. BDNF, NGF, CGRP, and Substance P were measured in the serum, peritoneal fluid, and DRG samples using four sandwich ELISA kits (Enzyme‐linked Immunosorbent, Bioassay Technology Laboratory; China: a. BDNF Assay Kit: Assay range 0.050–10 ng/ml and Sensitivity 0.01 ng/ml; b. NGF Assay Kit: Assay range 10–3000 ng/L and Sensitivity 5.01 ng/L; c. Substance P Assay Kit: Assay range 5–1000 ng/L and Sensitivity 2.49 ng/L; d. CGRP Assay Kit: Assay range 2–600 pg/ml and Sensitivity 1.02 pg/ml).

### 2.6 Statistical analysis

The SPSS software version 22 was used for data analysis. The data were analyzed using the one‐way ANOVA, repeated measures ANOVA, and Tukey post‐hoc test. *p* < 0.05 were considered significant. Data are shown as mean ± SEM.

## 3 RESULTS

### 3.1 Macroscopic and histopathological evaluation of endometriotic lesions

**FIGURE 2:**
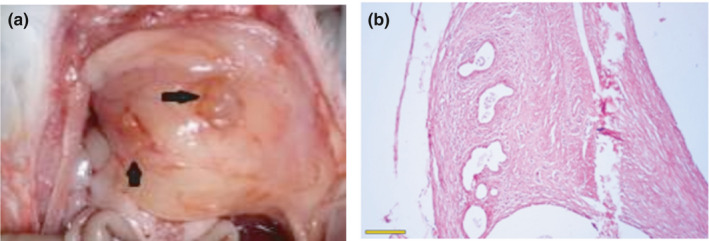
The representation of the experimental model of the endometriosis in rats: cyst formation (a); and histological view of the endometriosis in the abdominal wall in day 21 (b). The scale bar length is 300 µm

Endometriosis cyst formation was observed 21 days after endometriosis induction (Figure 2a) and histopathological examination revealed the presence of gland‐like structures resembling human endometriotic lesions confirming the successful implantation (Figure 2b).

### 3.2 Evaluation of endometriotic lesion size

**FIGURE 3:**
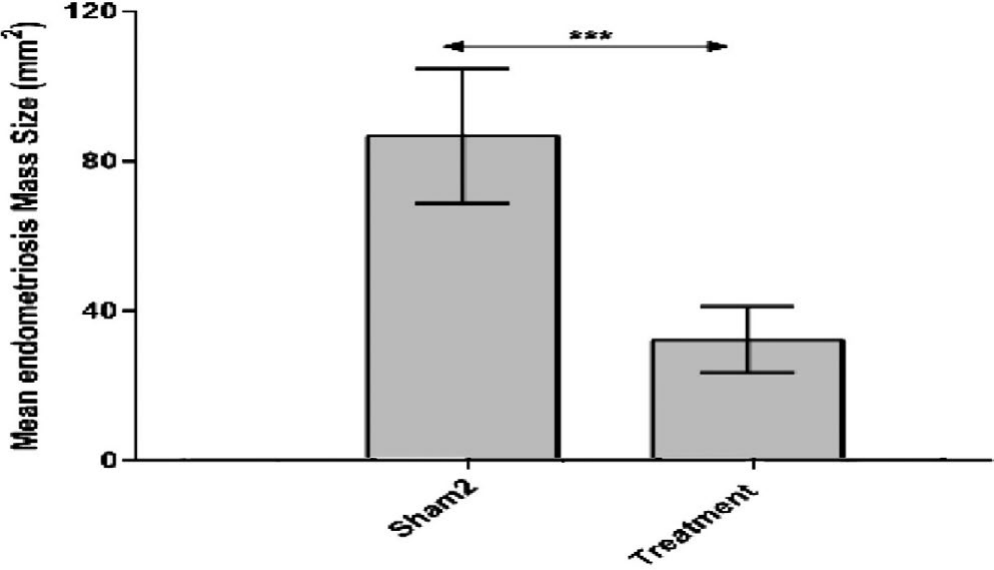
The mean mass sizes in the treatment and sham‐2 groups *** *p*<0.001

The results indicated that treatment with RJ significantly decreased the size of endometriotic lesions in the treatment group compared to the sham‐2 group (*p* < 0.001) (Figure 3).

### 3.3 Evaluation of weight and pain behavior

**FIGURE 4:**
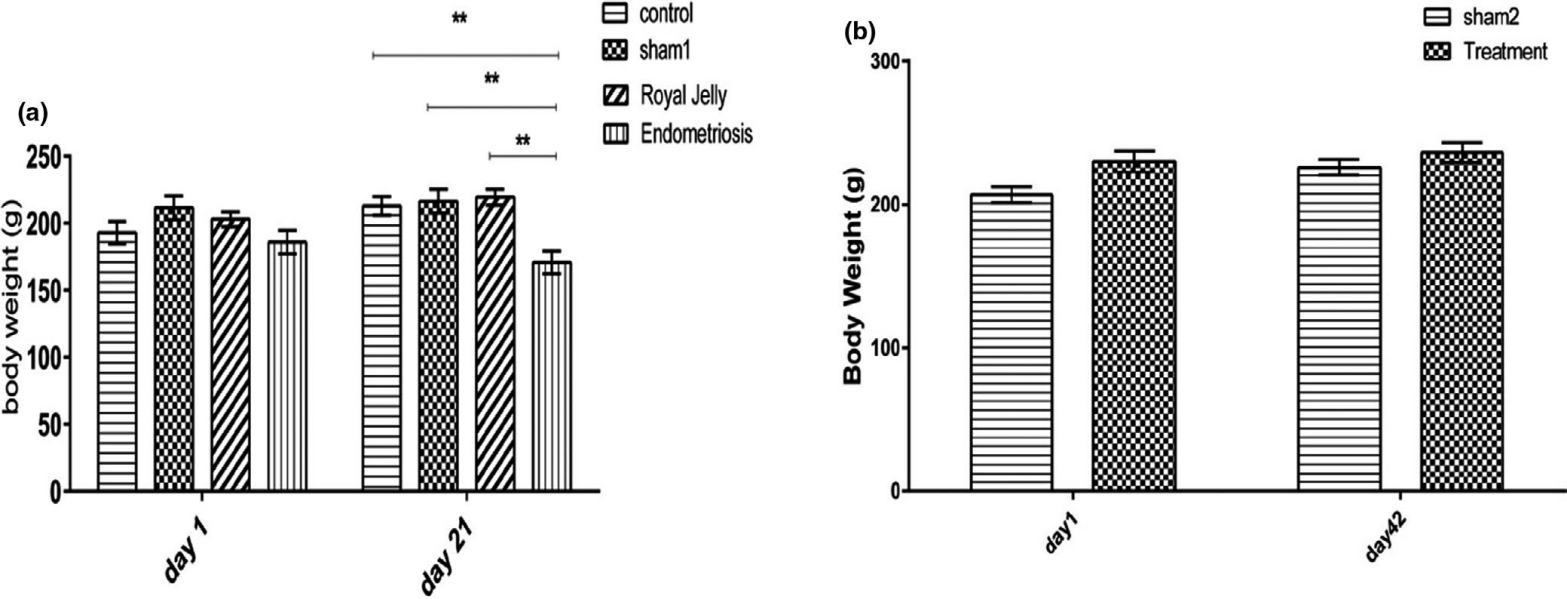
Comparison of weight alterations between the groups in phases one (a) and two (b): ** *p* < 0.01

Endometriosis induction significantly decreased the weights (*p* < 0.01) of rats in the endometriosis group compared to the other groups in phase one. On the other hand, between‐group or intra‐group weight alterations were not significant in phase two (*p* > 0.05) (Figure 4a,b).

**FIGURE 5:**
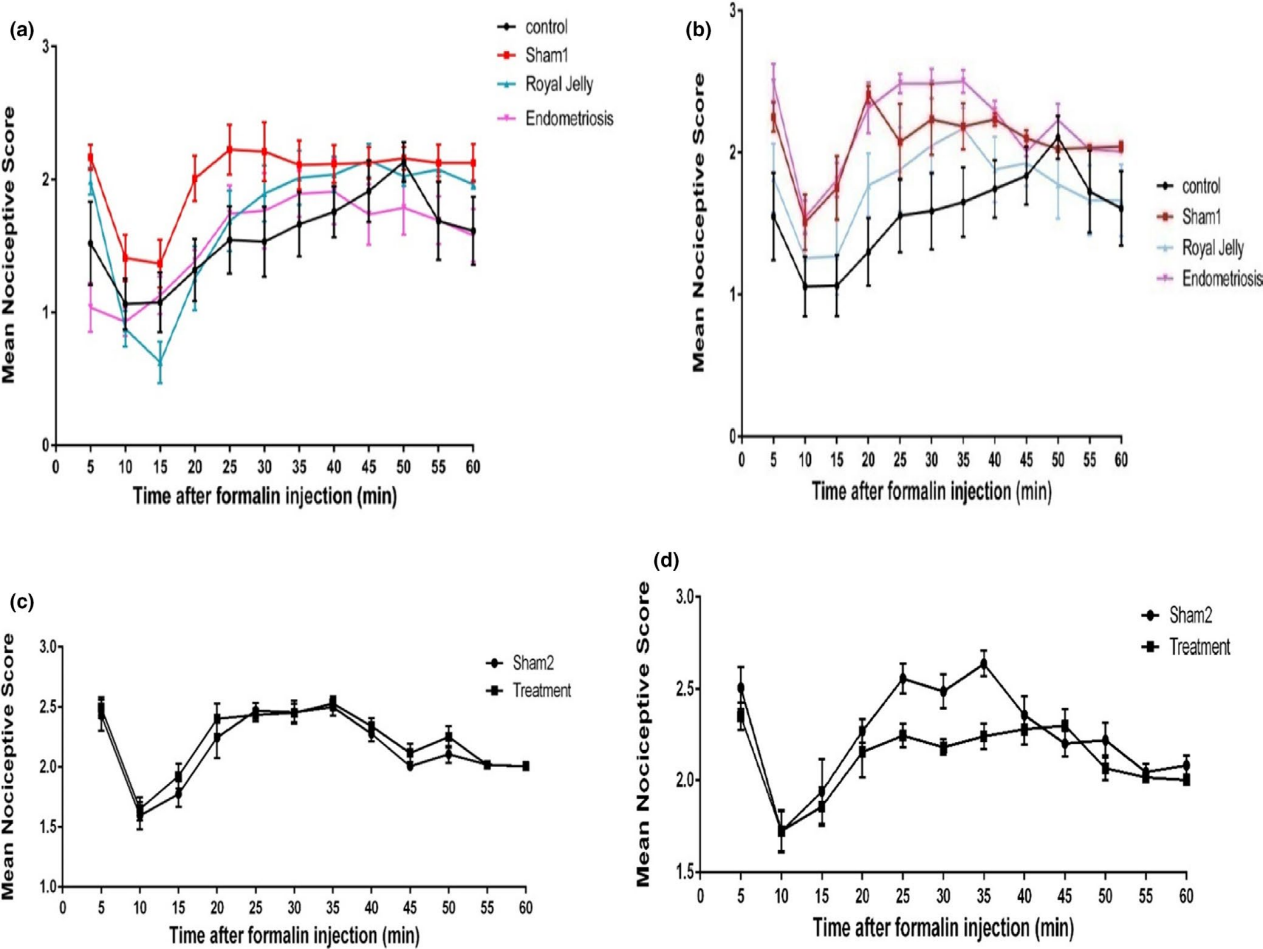
Comparison of the mean pain behavior scores on the first (a, c) and last days (b, d) of the study between groups in phases one and two

Regarding the mean pain behavior score in phase one (on day 21), the highest pain scores related to all three phases of formalin test were seen in the endometriosis (*p* < 0.01) and sham‐1 (*p* < 0.05) groups (Figure 5a,b). In phase two, administration of RJ for 21 days could significantly decrease the mean pain score in the late phase of the formalin test (*p* < 0.05) (Figure 5c,d).

### 3.4 Evaluation of BDNF, NGF, substance P, and CGRP

**FIGURE 6:**
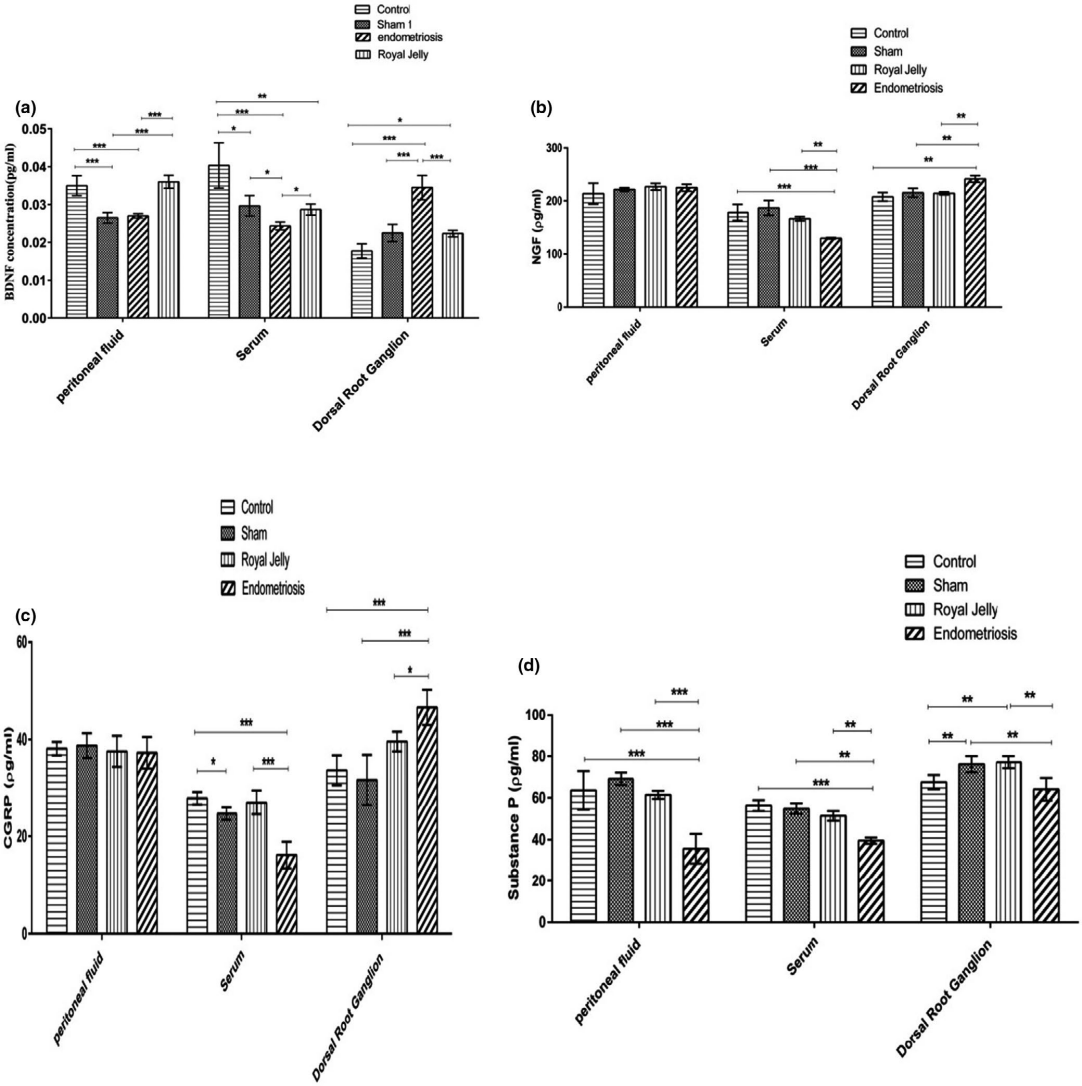
The means of BDNF (a), NGF(b), CGRP (c), and substance P (d) between groups in phase 1: *p < 0.05, **p < 0.01, ***p < 0.001

Comparison of the concentrations of neurotrophic factors between groups in phase one showed the highest levels of DRG‐BDNF (*p *< 0.001) and NGF (*p *< 0.01) and the lowest levels of serum‐NGF (*p *< 0.01) in the endometriosis group. Both serum and peritoneal fluid BDNF concentrations were also significantly lower in the endometriosis group compared to control and RJ groups (*p *< 0.05) (Figure 6a,b). Analysis of data related to CGRP showed the same pattern; the endometriosis group had the highest concentration of DRG‐CGRP (*p* < 0.05) and a lower serum concentration of CGRP compared to the control (*p *< 0.001) and RJ groups (*p *< 0.001) (Figure 6c). Among all phase one groups, the endometriosis group had the lowest values of substance P in the PF, serum, and DRG (Figure 6d).

**FIGURE 7:**
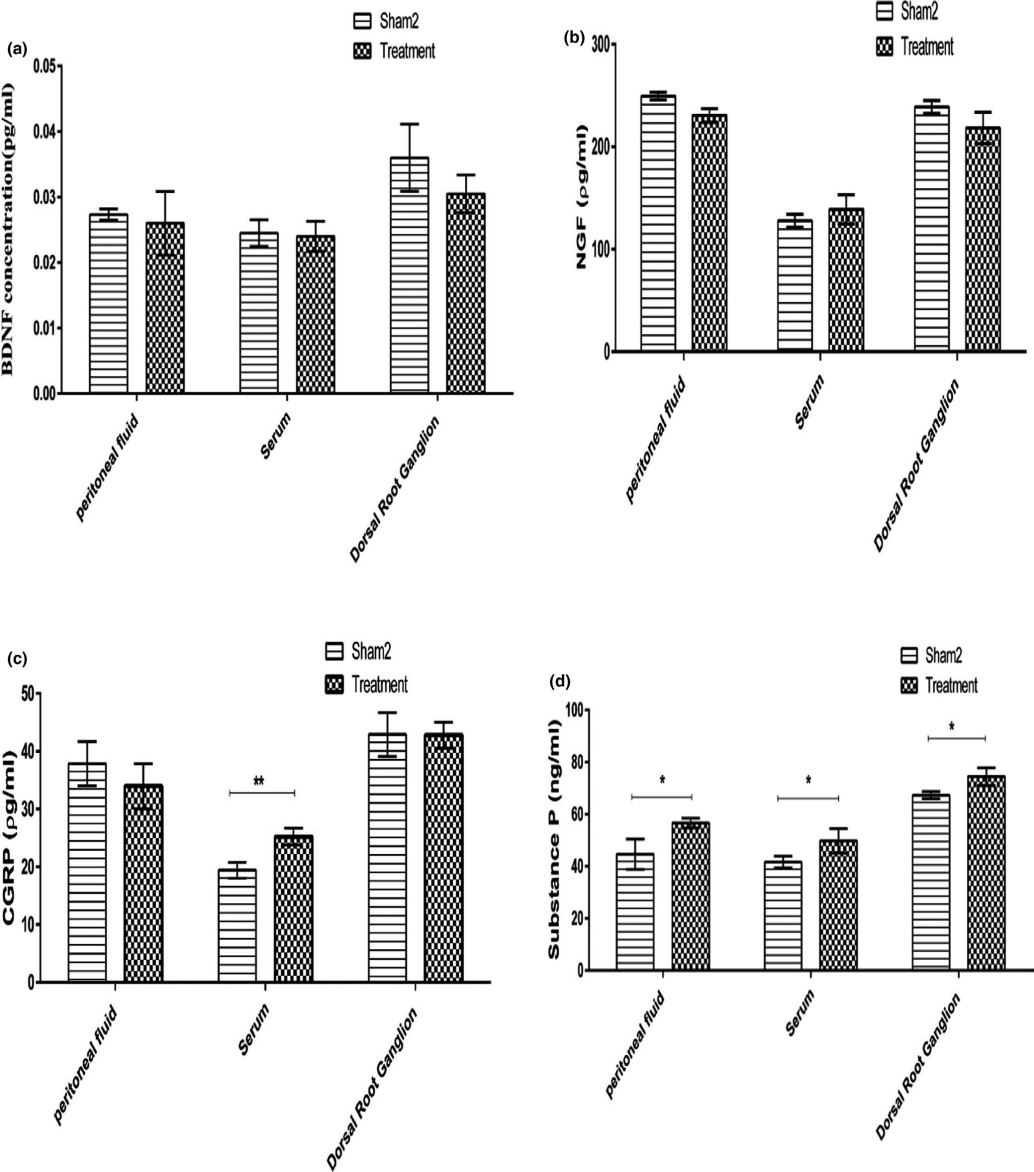
The means of BDNF (a), NGF (b), CGRP (c), and substance P (d) between groups in phase 2: *p < 0.05, **p < 0.01

Comparison of factors between groups in phase two showed no significant differences in the levels of BDNF & NGF in serum, PF, and DRG between groups (*p* > 0.05) (Figure 7a,b). However, the mean CGRP level in the serum as well as the mean substance P level in the DRG, serum, and PF was significantly higher in the treatment group compared to the sham‐2 group (*p *< 0.01) (Figure 7c,d).

## 4 DISCUSSION

Endometriosis patients search for complementary and alternative medicine with few adverse effects to relieve their symptoms. The goal of the present study was to evaluate the possible therapeutic effects of royal jelly (RJ) on endometriosis symptoms which have not been conducted previously in other investigations. Moreover, the correlation between endometriosis pain and neurotrophic factors was assessed.

According to the results, treatment with RJ for 21 days in phase two significantly decreased the size of endometriotic lesions. This effect of RJ may be related to its phenolic compounds with anti‐inflammatory, wound healing, anticancer, and vasodilatory properties (Viuda‐Martos et al., 2008). RJ flavonoids have antioxidant effects that can also influence the cell signaling pathways, inhibition of cell proliferation, and cell cycle arrest, which causes apoptosis in endometriotic lesions and reduces their size (Premratanachai & Chanchao, 2014). Although we could not find any other studies assessing the effects of RJ on the endometriotic lesion size, Shuang et al. (2017) found that treatment with RJ significantly controlled and inhibited the growth of breast cancer tumors through immunomodulation and antioxidant effects in mice. Treatment with 100, 200, and 300 mg/kg RJ for 13–17 days also significantly decreased the size of fibrosarcoma in mice (Shirzad et al., 2013).

The results showed that treatment with RJ for 21 days in phase two significantly decreased the mean pain score in the treatment group. This analgesic effect of RJ may be due to its antioxidant and neuroprotective properties. Moreover, RJ inhibits oxygenase and lipoxygenase activity. These enzymes are mainly involved in hyperalgesia related to endometriosis as a chronic inflammatory disease (Viuda‐Martos et al., 2008). In accordance with our results, Arzi, Houshmand, et al. (2015)). found that administration of 200 mg/kg RJ decreased the mean pain score (formalin test) equal to the administration of 300 mg/kg aspirin in rats. It should be also noticed that we could not confirm the analgesic effects of RJ when we compared the mean pain score between the RJ and control groups in phase one. RJ probably affects pain sensitivity when there is inflammation but it has no particular effect under normal conditions.

The present study found a significant increase in the pain score of the formalin test in the endometriosis and sham‐1 groups after 21 days compared to other groups. Moreover, the results of the pain score on days 1 and 21 revealed a significantly higher score in the endometriosis group on day 21. According to former studies in animal models, several processes including hyperinflammatory responses in the peritoneal cavity, peritoneal adhesion, formation of scar tissue, extracellular matrix degradation, angiogenesis, and neurogenesis happen with a successful establishment of endometriosis. These processes may contribute to hyperalgesia as the main symptom of endometriosis (Bruner‐Tran et al., 2018). Furthermore, endometriosis by central sensitization can increase sensitivity to subsequent painful stimuli and cause hyperalgesia and allodynia in endometriosis‐associated pain (McKinnon et al., 2015; Stratton & Berkley, 2011). Hernandez et al. (2015) by using a hot plate test showed hyperalgesia 14 days post‐endometriosis induction. It has been also shown a significant decrease in the tail‐flick latency in an endometriosis model group compared to the control group (Lian et al., 2017). It should be also noted that the increase in the pain scores in the sham‐1 group in our study may confirm the hypothesis that abdominal and pelvic surgical operations can increase the risk of endometriosis (Liu et al., 2016).

According to the results, significant increases of DRG‐NGF and BDNF were observed in the endometriosis group compared to the other groups in phase one. These results may confirm that endometriosis induction could lead to hyperinnervation, pain, and overexpression of neurotrophic factors like BDNF and NGF in the first station of the nociceptive nerve cells in DRG (Kobayashi et al., 2014). Huang (2018) also showed significant increases of BDNF expression and BDNF receptors in DRG of endometriosis‐like diseases those are accompanied by complex mechanisms of pain (increased cytokines, peripheral and central nerve sensitization, and ischemia). Dewanto et al. (2016) showed that in a mouse model of endometriosis, NGF and BDNF had a notable role in the DRG‐sensory nerve development, axonal branching, and elongation. The results of a mouse model study by Li et al. (2011) also indicated increased NGF levels in the DRG of the endometriosis group compared to the controls.

In phase one, DRG‐CGRP was significantly higher in the endometriosis group compared to other groups. Similarly, Lian et al. (2017). using western blot and RT‐qPCR analyses showed that CGRP protein in DRG of the endometriosis group was significantly higher than the sham group.

The results showed significant decreases in the serum and PF‐BDNF, serum‐NGF, and CGRP in the endometriosis group compared to other groups in phase one. Previous studies reported different findings (increase, decrease, or no difference) regarding NGF, BDNF, and CGRP alterations in the serum or PF in the endometriosis group (Arellano et al., 2013; Ding et al., 2018; Qin et al., 2019). Perricos et al. (2018) found that alterations of serum NTs may be specific to the endometriosis stage/lesion type rather than its general feature. Dewanto et al. (2016) identified that BDNF and NGF receptors (TrkA and B) in the peritoneum have high affinity for NTs bonding resulting in localization of in the endometriotic lesions. Furthermore, Tokushige et al. (2006). observed a significant increase in the CGRP nerve fibers in the endometriotic lesions. This accumulation of CGRP in the endometriotic lesions may describe the low level of CGRP in serum.

Our results showed that of all groups in phase one, the endometriosis group had the lowest concentrations of substance P in serum, peritoneal fluid, and DRGs. It is supposed that the increase of such a pro‐inflammatory factor might be time‐dependent (Chiantera et al., 2017). Compatible with our results, Medina et al. showed substance P in the uterine endometrial and myometrial layers of both subjects with and without endometriosis. They also reported that substance P with contractility properties had a possible role in pain associated with other physiologic or pathologic conditions like dysmenorrheal and so on (Medina & Lebovic, 2009). Sanfilippo et al. (1992) showed no significant difference in the level of substance P between endometriosis patients and the controls. They concluded that as substance P naturally is present in the peritoneal fluid; its concentrations may not be significantly affected by endometriosis or pelvic adhesions.

The present study found no significant alterations in the neurotrophic factors between the study groups in phase two. It seems that several factors like decreased size of endometriotic lesion following RJ treatment or time/dose‐dependency of RJ administration may be involved in such responses.

Although no significant difference was found in neurotrophic factors between the study groups in phase two, DRG, serum, and PF substance P and serum CGRP were significantly higher in the treatment group compared to the sham‐2 group. It seems that these findings may be related to phenolic compounds of RJ, such as flavonoids, which have estrogenic effects. It has been reported that the phytoestrogenic composition of RJ competes with estrogens for binding to the estrogen receptors (Mishima et al., 2005). Estrogenic factors can upregulate SP and CGRP and their receptors in visceral pain sensitivity (Liang et al., 2018). These responses are dose‐dependent (Mowa et al., 2003).

Finally, the results showed that endometriosis induction significantly decreased the weight of animals in phase one. This weight loss pattern may be related to an increase in the serum and PF leptin levels (an anti‐hunger hormone) observed in endometriosis patients (Matarese et al., 2000). Moreover, overexpression of several genes including Cyp2r1, Fabp4, Mrc1, and Rock2 has been shown in the liver and visceral adipose tissue of endometriosis cases. These genes with increase of insulin sensitivity and lipid metabolism, as well as, anorexigenic effects may protect endometriosis patient against obesity (Goetz et al., 2016).

## 5 CONCLUSION

Induction of endometriosis leads to hyperalgesia, which may be associated with a significant increase in NGF, BDNF, and CGRP levels in DRG. Treatment with RJ could decrease the size of endometriotic lesions as well as pain scores; however, these therapeutic effects were not associated with alterations in the levels of NTs. The findings may shed light on other complementary and alternative remedies for endometriosis.

## CONFLICT OF INTEREST

The authors declare no conflict of interest.

## AUTHOR CONTRIBUTIONS

Dr. Taherianfard and Dr. Farahani carried out the design, data acquisition, and coordinated the study. Dr. Naderi, Dr. Ferrero, and Dr. Farahani coordinated and carried out all the experiments. Dr. Taherianfard analyzed data. Dr. Taherianfard, Dr. Ferrero, and Dr. Farahani participated in manuscript preparation. All authors have read and approved the content of the manuscript and agree to be accountable for all aspects of the work in ensuring that questions related to the accuracy or integrity of any part of the work are appropriately investigated and resolved.

## ETHICAL STATEMENT

This animal study was conducted in compliance with the procedures confirmed by the Ethical Committee for Animal Experiments at Shiraz University.
